# What is the relationship between renal function and visit-to-visit blood pressure variability in primary care? Retrospective cohort study from routinely collected healthcare data

**DOI:** 10.1136/bmjopen-2015-010702

**Published:** 2016-06-10

**Authors:** Daniel S Lasserson, Nynke Scherpbier de Haan, Wim de Grauw, Mark van der Wel, Jack F Wetzels, Christopher A O'Callaghan

**Affiliations:** 1 Nuffield Department of Medicine, University of Oxford, Oxford, UK; 2 Department of Geratology, NIHR Oxford Biomedical Research Centre, Oxford University Hospitals NHS Trust, Oxford, UK; 3 Department of Primary and Community Care, Radboud University Nijmegen Medical Centre, Nijmegen, The Netherlands; 4 Department of Nephrology, Radboud University Nijmegen Medical Centre, Nijmegen, The Netherlands

**Keywords:** Blood Pressure, Chronic kidney disease, Variability

## Abstract

**Objective:**

To determine the relationship between renal function and visit-to-visit blood pressure (BP) variability in a cohort of primary care patients.

**Design:**

Retrospective cohort study from routinely collected healthcare data.

**Setting:**

Primary care in Nijmegen, the Netherlands, from 2007 to 2012.

**Participants:**

19 175 patients who had a measure of renal function, and 7 separate visits with BP readings in the primary care record.

**Outcome measures:**

Visit-to-visit variability in systolic BP, calculated from the first 7 office measurements, including SD, successive variation, absolute real variation and metrics of variability shown to be independent of mean. Multiple linear regression was used to analyse the influence of estimated glomerular filtration rate (eGFR) on BP variability measures with adjustment for age, sex, diabetes, mean BP, proteinuria, cardiovascular disease, time interval between measures and antihypertensive use.

**Results:**

In the patient cohort, 57% were women, mean (SD) age was 65.5 (12.3) years, mean (SD) eGFR was 75.6 (18.0) mL/min/1.73m^2^ and SD systolic BP 148.3 (21.4) mm Hg. All BP variability measures were negatively correlated with eGFR and positively correlated with age. However, multiple linear regressions demonstrated consistent, small magnitude negative relationships between eGFR and all measures of BP variability adjusting for confounding variables.

**Conclusions:**

Worsening renal function is associated with small increases in measures of visit-to-visit BP variability after adjustment for confounding factors. This is seen across the spectrum of renal function in the population, and provides a mechanism whereby chronic kidney disease may raise the risk of cardiovascular events.

**Strengths and limitations of this study**

- We studied blood pressure (BP) variability in a patient cohort generalisable to primary care where the majority of patients with chronic kidney disease are diagnosed and monitored, as previously BP variability in chronic kidney disease (CKD) has only been studied in patients with advanced renal disease under specialist care.
- We examined measures of BP variability that are independent of mean BP, which have been shown to have greater prognostic significance than mean BP alone.
- We were not able to assess the effect of different exposures to antihypertensive medication.
- We did not have access to data on cardiovascular outcomes to test the clinical significance of visit-to-visit variability in this patient cohort.

## Introduction

Hypertension is a major vascular risk factor, and average blood pressure (BP) correlates with cardiovascular disease and death.^1^ Recently, visit-to-visit variability in BP measurements has been shown to convey an additional independent risk for cardiovascular events and to be associated with all-cause mortality in the general population.^2^ ^3^ BP variability is reproducible within individuals over time in clinical trials^4^ and in observational data from routine care.^4^ The cardiovascular prognostic significance of BP variability is increasingly recognised, and the variability is not explained by poor medication adherence.^5^

Increased BP variability has been observed in small groups of patients with relatively advanced renal disease under specialist hospital care. In a small hospital cohort, visit-to-visit BP variability was independently associated with albuminuria and with increased renal vascular resistance as assessed by Doppler ultrasound.^6^ A study of 56 patients with non-diabetic chronic kidney disease (CKD) indicated a correlation between indices of BP variability and the rate of decline in renal function.^7^ In type 2 diabetes, visit-to-visit variability in systolic BP correlates with the level of albuminuria, ankle brachial pressure index,^8^ and the probability of developing new albuminuria.^9^ In a set of secondary care patients with substantial renal impairment, there was a correlation between BP variability and a composite outcome of death and cardiovascular events.^10^ In patients on haemodialysis, visit-to-visit variability in BP is an independent predictor of vascular events, but this is complicated by the dependence of BP in this context on intradialytic fluid gain.^11^

However, as BP variability increases with mean BP, and renal disease is associated with increased mean BP, the relationship between BP variability and renal impairment can only be established using metrics of BP variability that have been shown to be independent of mean BP.^12^ None of the renal studies mentioned above reported statistics of BP variability that were demonstrated to be independent of the mean BP and, therefore, an evidence gap remains concerning whether declining renal function is associated with BP variability.

It is important to establish whether there is a real relationship between renal impairment and increased BP variability because the link might be causal and have important implications for therapy in CKD. Analysis of large sets of routinely collected healthcare data has demonstrated that CKD is an important independent risk factor for cardiovascular disease even after controlling for other known risk factors.^13^ ^14^ The prevalence of CKD is around 5–10%, and the majority of patients with CKD are managed by non-specialists or primary care physicians.^15^ ^16^ Given that a substantial proportion of the population have early stage CKD, it is therefore crucial to establish whether early stage CKD is associated with increased BP variability, as there could be major implications for population cardiovascular risk factor monitoring and treatment.

We therefore sought to determine whether renal function was associated with visit-to-visit BP variability using a large dataset of routinely collected healthcare data.

## Methods

We retrospectively defined a patient cohort (age ≥18 years) using routinely collected healthcare data from 47 primary care practices in the Nijmegen region, the Netherlands, with a combined registered population of 207 468 people, as part of the CONTACT study (Consultation of Nephrology by Telenephrology Allows optimal Chronic kidney disease Treatment in primary care), Netherlands Trial Registration code 2368, with approval from the Medical Research Ethics Committee Arnhem/Nijmegen, registration number 2010/187.

Data included demographic details, medical history with conditions diagnosed in the course of routine clinical practice, BP measurements, antihypertensive prescribing and renal function between 1 January 2008 and 1 January 2011. We did not seek to include patients receiving dialysis in this study population. The Chronic Kidney Disease Epidemiology Collaboration (CKD-EPI) formula was used to calculate estimated glomerular filtration rates (eGFRs) using the first creatinine measurements in the time period, which were either standardised to isotope dilution-mass spectrometry (IDMS), or subject to the appropriate correction factor for laboratories using the Jaffé technique.^17^ CKD-EPI eGFRs have been shown to correlate better with measured GFRs than eGFRs obtained with the Modification of Diet in Renal Disease (MDRD) study formula.^18^ The first seven office BP measurements were used to calculate variability metrics, as this approach has been shown to optimise reproducibility.^12^ Dutch primary care physicians and practice nurses have free access to the Dutch College of General Practitioners guideline on cardiovascular risk management for a clear description of standardised office BP measurement, which is in agreement with international standards.

Given that blood pressure variability is associated with mean BP, we calculated BP variability independent of mean (VIM) BP using standard formulae for measures that have prognostic significance for cardiovascular events.^12^ For the systolic and diastolic BP of individuals with at least seven BP measures, we calculated the SD, successive variation (SV), average real variability (ARV), and transformed these to be independent of mean BP^12^ (see online supplementary information for formulae). Observed associations between renal function and BP variability (systolic BP metrics described above and also including maximum systolic BP), may be confounded by other predictors of variability, and we therefore tested the association between renal function and measures of BP variability using multiple stepwise linear regression adjusting for age, sex, mean BP, diabetes, history of cardiovascular disease (including vascular events, arrhythmia and heart failure), proteinuria, time interval between BP measures and class of antihypertensive drug prescribed. We included mean BP as a covariate to ensure that the effect of mean BP was truly adjusted for in regressions testing the relationship between VIM statistics and eGFR. Analyses were carried out using R (V.3.0.1) and SPSS V.21.

10.1136/bmjopen-2015-010702.supp1 Supplementary data

## Results

Among the 207 468 total registered population, 162 562 were over the age of 18 years, of whom 63 073 (39%) had renal function measured during the study period. Of these, 19 175 (30%) had at least seven BP measurements recorded at different visits. Table 1 lists the features of the group which had a mean age of 65.5 years, a mean blood creatinine concentration of 85.3 µmol/L (0.97 mg/dL), a mean eGFR of 75.6 mL/min/1.73 m^2^ and a mean systolic BP of 148.3 mm Hg.

**Table 1:** Demographics of population studied (n=19 175)

Age	65.5 (12.3) years
% Female	57%
CKD-EPI eGFR	75.6 (18.0) mL/min/1.73 m^2^
Creatinine	85.3 (23.0) µmol/L or 0.97 (0.26) mg/dL
Diagnosed with diabetes	37.5%
Number of antihypertensive drugs prescribed (%, proportion of patients)	0–16.5
	1–26.7
	2–30.1
	3–18.8
	4–6.8
	5–1.0
	6–0.1
Interval between 1st and 7th BP measure	627 (295) days
Systolic BP at baseline	148.3 (21.4) mm Hg

Continuous data are presented as mean (SD).

BP, blood pressure; CKD-EPI, Chronic Kidney Disease Epidemiology Collaboration formula; eGFR, estimated glomerular filtration rate.

The mean time interval from the first to the final seventh BP measurement was 22 months (SD 11 months). Mean systolic BP rose with age (R=0.19, p<0.001) and with falling eGFR (R=−0.11, p<0.001). Correlation analysis of transformed BP variability measures was consistent with their independence from mean BP (R values; SD independent of mean (SDIM) −0.009, p=0.23; SV independent of mean (SVIM) −0.009, p=0.22; ARV independent of mean (ARVIM) −0.01, p=0.15). Online supplementary table S1 shows the results of curve fitting to generate the transformed variables.

Table 2 shows the correlations between mean systolic BP and its variability with deteriorating eGFR and with increasing age. All measures of intraindividual BP variability increase progressively with declining eGFR and with increasing age.

**Table 2:** Correlation of systolic BP metrics with eGFR and with age

Systolic BP metric	Correlation with eGFR	Correlation with age
Mean	−0.110***	0.190***
Maximum	−0.121***	0.178***
SD	−0.116***	0.115***
SV	−0.115***	0.131***
ARV	−0.113***	0.129***
SDIM	−0.086***	0.054***
SVIM BP	−0.088***	0.076***
ARVIM BP	−0.086***	0.075***

***p<0.001.

ARV, average real variability; ARVIM, ARV independent of mean; BP, blood pressure; eGFR, estimated glomerular filtration rate; SDIM, SD independent of mean; SV, successive variation; SVIM, SV independent of mean.

**Figure 1:**
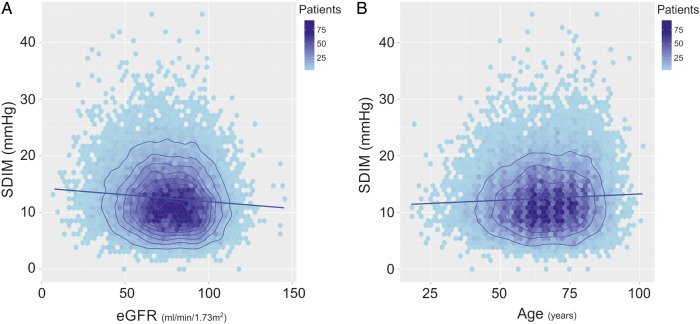
Renal function and age, both associated with blood pressure variability. Colours indicate the number of data points in each shaded area of the plot. The number of patients represented by each coloured polygon, that is, the data frequency, is indicated by the colour scale. Contours are added to aid visualisation of the shape of the frequency distribution across the two-dimensional plot. The contours are derived from kernel density estimation of the data frequency, and represent the boundaries of intervals of equal magnitude in the data frequency. The regression line is shown. (A) The relationship between SD independent of mean (SDIM) and estimated glomerular filtration rate (eGFR). (B) The relationship between SDIM and age.

The relationships of eGFR and age with SDIM, as an exemplar of the associations with VIM, are shown in figure 1A, B.

Multiple stepwise regression analysis was performed to examine the association between renal function and BP variability. Table 3 shows the standardised β coefficients and associated p values from multiple regression analyses of eGFR on BP variability metrics using, age, sex, mean systolic BP, diabetes, cardiovascular disease, proteinuria, interval between BP measures and the class of antihypertensive drug prescribed as covariates (medication classes are not shown, see online supplementary table S2). Non-standardised coefficients for categorical variables are shown in online supplementary table S3).

**Table 3:** Standardised β coefficients and associated p values from multiple linear regressions of eGFR on measures of BP variability adjusted for potential confounders (medication classes not shown)

Measure of BP Variability	eGFR Std β	Age Std β	Sex Std β	Mean BP Std β	Vascular disease Std β	BP interval Std β	Diabetes Std β
SD	−0.04***	−	0.06***	0.37***	0.07***	−	−0.06***
ARV	−0.04***	0.02***	0.06***	0.31***	0.06***	0.02*	−0.035***
SRV	−0.04***	0.02*	0.07***	0.32***	0.06***	0.02*	−0.03***
Max systolic	−0.02***	−0.01*	0.03***	0.88***	0.03***	−	−0.03***
SDIM	−0.05***	−	0.07***	−0.04***	0.08***	−	−0.07***
ARVIM	−0.04***	0.03*	0.06***	−0.04***	0.07***	0.02*	−0.04***
SRVIM	−0.05***	0.03**	0.07***	−0.04***	0.07***	0.02*	−0.041***

*p<0.05, **p<0.01, ***p<0.001.

ARV, average real variability; ARVIM, ARV independent of mean; BP, blood pressure; eGFR, estimated glomerular filtration rate; SDIM, SD independent of mean; SRV, successive real variability; SRVIM, SRV independent of mean.

Coefficients shown if included in stepwise multiple regression.

Lower eGFR was consistently associated with increasing measures of BP variability (both with and without transformation to be independent of mean BP), as evidenced by the significant negative standardised β coefficients across the regressions in the presence of potential confounders. Although the presence of proteinuria was not associated with any measure of BP variability, a history of cardiovascular disease was positively associated with increased BP variability. All classes of antihypertensive medication were positively associated with measures of BP variability where significant relationships were observed (see online supplementary table S2).

Greater BP variability was associated with longer time intervals between BP measures, as evidenced by the positive coefficients where significant relationships were observed.

## Discussion

Both mean BP and visit-to-visit BP variability increased with age and with worsening renal function in this large community-based population. Further analysis with multiple linear regression demonstrated that worsening renal function remains significantly associated with increased BPVIM, even after adjustment for age, sex, diabetes, history of cardiovascular disease, mean systolic blood pressure, interval between BP measures, proteinuria and the class of antihypertensive drug prescribed. This is important because visit-to-visit BP variability over the medium term is a strong predictor of cardiovascular outcome independently of mean BP.^3^ In addition, this relationship between renal function and BP variability may explain, in part, the association between renal function and cardiovascular disease, which remains even after controlling for other established cardiovascular risk factors.^15^

Hitherto, the relationship between renal disease and BP variability has not been studied in large unselected community-based populations. In specialist services which typically care for patients with advanced renal disease, or those requiring renal replacement therapy, visit-to-visit variability in BP has been linked to poorer renal^6^ and cardiovascular outcomes.^19^ In settings, such as the UK or the Netherlands, where guidelines exist for specialist referral,^20^ ^21^ there is substantial enrichment in specialist service populations for advanced CKD stage, rapidly declining renal function, or significant proteinuria. Observational studies of these groups for prognostic factors has an uncertain generalisability to the much larger, but less severely affected, population monitored predominantly by non-specialists or primary care physicians. Furthermore, none of the previous studies of renal disease have used metrics of BP variability that have been shown to be independent of mean BP, and so, do not clearly distinguish between the effects of raised systolic BP and BP variability.

The results of the regression analyses showed a consistent positive relationship between the presence of cardiovascular disease and BP variability, a finding that is consistent with the known prognostic significance of BP variability.^22^ Importantly, the time interval between BP measures was positively associated with BP variability, indicating that longer durations between measures were associated with greater variability. This counters the hypothesis that associations between greater BP variability and reduced renal function are an artefact of acute illness where clinical monitoring will be more frequent, or due to increased monitoring at the time of initiation of antihypertensive drugs. If acute illness or the initiation of antihypertensive therapy was responsible for increasing BP variability, one would expect greater variability to be associated with shorter time intervals between BP measures in a period of clinical instability or medication change. We presented standardised coefficients for all included variables in order to facilitate a clear clinical interpretation of the relative contributions of the predictors of the BP variability measures.

A limited *post hoc* analysis of around 2000 patients selected for the ASCOT-BPLA trial showed a weak relationship between blood creatinine and BP variability, but the trial design undermines the generalisability of this analysis to representative patient populations.^22^ The study excluded anyone with a creatinine of >200 µmol/L (2.26 mg/dL), with clinically important renal disease, with secondary hypertension (which could include renal disease), or any concomitant disease requiring calcium channel blockers, angiotensin converting enzyme inhibitors, β blockers, or diuretics. These criteria would likely exclude the majority of patients with chronic kidney disease. Further, the study excluded anyone <55 years of age, eGFRs were not reported, and it is unclear whether creatinine assays were standardised to IDMS values. Creatinine is influenced by both age and renal function—in part because of age-related changes in muscle mass—and the analysis was weakened in this older trial population by the comparison of creatinine values across different ages.

The relationship we observed between CKD and BP variability in a wider population demonstrates that, even in the early stages of renal dysfunction, BP variability is present at a level associated with significant cardiovascular risk. The measures of variability (ARV, SV and those transformed to be independent of mean) in eGFR ranges corresponding to CKD stage 4 in this study are similar to those seen in cohorts of patients with transient ischaemic attack (TIA) in the UK-TIA trial and the European Carotid Surgery Trial.^12^ Furthermore, the timeframe of BP variability measurement in this study (mean time between BP measures of 104 days) is consistent with the time frames between repeat BP measures during follow-up in the trials establishing increased BP variability and its prognostic significance.^12^ ^22^

Our study has some limitations. The population studied exists in a healthcare context where the value of treating hypertension is recognised, and this is likely to reduce the slope of the relationship between mean systolic BP and eGFR. In addition, the variability associated with the estimation of renal function,^16^ and intrinsic error will result in regression dilution bias. However, this will bias the study towards the null hypothesis, such that the strength of the relationship between renal function and BP variability will be underestimated rather than overestimated. Error associated with the measurement of BP variability will widen the confidence limits associated with the relationship. Furthermore, while our adjustment for antihypertensive use included drug-class specific effects, we were not able to assess the effect of exposure by examining drop-in and drop-out of treatment, but given that variability may be affected by some drugs more than others,^23^ this constraint is also likely to reduce rather than enhance the associations that we have found. The findings are limited to patients with multiple BP readings taken at their general practice, and at least one measure of renal function. Nevertheless, despite these limitations, we have found consistent and significant associations between measures of renal function and BP variability.

Our results demonstrate that the relationship between eGFR and BP variability exists across the spectrum of eGFR which corresponds to different stages of CKD. This relationship is consistent with a causative relationship between diminished eGFR and BP variability, but causation cannot be established in an observational study. Nevertheless, this may prove to have clinical usefulness in determining vascular risk and prognosis, as we have demonstrated that BP variability is readily detectable and quantifiable in retrospective data in the non-specialist setting.

Studies of the prognostic significance of BP variability in general CKD populations are required as monitoring BP variability may refine the prediction of cardiovascular risk. The correlations that we have identified are of small magnitude, but they are independent, and given the population prevalence of CKD, this correlation will have consequences for cardiovascular risk at the population level. Clinical trials are needed in CKD to establish the value or otherwise for renal and cardiovascular outcomes of tailoring antihypertensive therapy to minimise BP variability.
