# Supplementary material for: What is the relationship between renal function and visit-to-visit blood pressure variability in primary care? Retrospective cohort study from routinely collected healthcare data
Source: BMJ Open. 2016 Jun 10;6(6):e010702. doi: 10.1136/bmjopen-2015-010702 (PMC4908894; doi:10.1136/bmjopen-2015-010702)
Supplement: Supplementary data [file bmjopen-2015-010702supp.pdf]

Supplementary Information

Formulae for variability metrics

Different methods to measure variability have been used in the literature with the following being adopted by researchers in this field.

Successive variation (SV)

$$SV = \sqrt{(\frac{1}{n-1}\sum_{i=1}^{n-1}(BP_{i+1} - BP_i)^2)}$$

Where for each patient BP_i is the *i*th measure for I = 1,2….n

Average Real Variability (ARV)

$$ARV = \frac{1}{n-1}\sum_{i=1}^{n-1}|BP_{i+1} - BP_i\,|$$

Where for each patient BP_i is the *i*th measure for I = 1,2….n

Correlations between mean BP and measures of variability

The following transformations have been used to reduce the influence of mean BP on measures of variability thereby reducing the confounding from BP in associations between BP variability and cardiovascular events.(1)

SD independent of mean (SDIM)

$$SDIM = k\frac{\mathrm{SD}}{mean^x}$$

Where *x* is found by curve fitting for the entire patient cohort from a plot of SD versus mean BP (separately for systolic and diastolic), and k is an arbitrary constant such that the SDIM statistic is of the same order of magnitude as the SD statistic. These parameters are then applied to individual patient mean BP and SD to create the SDIM statistic.

SV independent of mean (SVIM)

$$SVIM = k\frac{\mathrm{SV}}{mean^x}$$

Where *x* is found by curve fitting for the entire patient cohort from a plot of SV versus mean BP (separately for systolic and diastolic), and k is an arbitrary constant such that the SVIM statistic is of the same order of magnitude as the SV statistic. These parameters are then applied to individual patient mean BP and SV to create the SVIM statistic.

ARV independent of mean (ARVIM)

$$ARVIM = k\frac{\text{ARV}}{mean^{x}}$$

Where *x* is found by curve fitting for the entire patient cohort from a plot of ARV versus mean BP (separately for systolic and diastolic), and k is an arbitrary constant such that the ARVIM statistic of the same order of magnitude as the ARV statistic. These parameters are then applied to individual patient mean BP and ARV to create the ARVIM statistic.

Supplementary Table 1. Values of x from Curve Fitting

Statistic	x
**SDIM**	**1.64**
**SRVIM**	**1.49**
**ARVIM**	**1.51**

The values of $\mathbf{x}$ are of a similar order of magnitude to those found in VIM analyses from trials recruiting patients at high cardiovascular risk. Specifically for SDIM, SRVIM and ARVIM the values of $\mathbf{x}$ were 1.67, 1.65 and 1.83 respectively in the UK TIA Trial and 1.78. 1.89 and 1.99 respectively in the European Carotid Surgery Trial.(1)

<u>Supplementary Table 2. Non-standardised beta coefficients and associated p values for drug classes as covariates in multivariable linear regression of eGFR on measures of BP variability</u>

**BP measure**	ACEI/ARB	Beta blockers	Thiazide like diuretics	Calcium channel blockers	Mineralocorticoid receptor blockers
**Maximum systolic BP**	0.76^{***}	0.57^{**}	0.43^{*}	0.46^{*}	1.17^{*}
**Average real variability**	0.56^{***}	0.53^{***}		0.37^{**}	
**Successive variation**	0.67^{***}	0.6^{***}		0.44^{**}	0.86^{*}
**Standard deviation of systolic BP**	0.59^{***}	0.36^{***}	0.33^{***}	0.31^{**}	1.27^{***}
**Average real variability independent of mean**	0.61^{***}	0.55^{***}		0.36^{**}	0.71^{*}
**Successive variation independent of mean**	0.75^{***}	0.64^{***}		0.43^{**}	1.01^{**}
**Standard deviation independent of mean**	0.63^{***}	0.39^{***}	0.37^{***}	0.29^{**}	1.39^{***}

$^{*}P<0.05$, $^{**}P<0.01$, $^{***}P<0.001$

Supplementary Table 3. Non-standardised beta coefficients for categorical variables included in the multiple regressions of eGFR on measures of BP variability

Measure of BP Variability	Sex<br><br>beta	Vascular disease<br><br>beta	Diabetes<br><br>beta
**SD**	0.674***	0.89***	-0.65***
**ARV**	0.77***	0.88***	-0.43***
**SRV**	0.98***	1.06***	-0.47***
**Max systolic**	1.15***	1.25***	-1.03***
**SDIM**	0.65***	0.91***	-0.67***
**ARVIM**	0.74***	0.87***	-0.51***
**SRVIM**	0.94***	1.07***	-0.55***

*P<0.05, **P<0.01, ***P<0.001

References

1. Howard SC, Rothwell PM. Reproducibility of measures of visit-to-visit variability in blood pressure after transient ischaemic attack or minor stroke. Cerebrovascular diseases (Basel, Switzerland). 2009;28(4):331-40.
